# Dental Fees

**Published:** 1873-09

**Authors:** 


### DENTAL FEES.

We hear much complaint of the inadequate fees obtained by dentists, and we propose to treat of the subject and to suggest a remedy. It is a noticeable fact that dentists receive the poorest fees of any of the professions. And the question arises, why is it so? One reason is because the profession as a class are not, strictly speaking, educated men. They are in too many instances men who have adopted the profession as a "dernier resort," and if they make journeymen's wages appear to be satisfied. We often see men who have devoted many years of their life to their profession receiving the pitiful sum of one dollar for filling a cavity which would cost the patient three were he to go to a city (we are speaking more particular of country towns,). And why does he accept this small sum? Simply because some dentist has started in his town and will not come up to the old practitioner's prices. Consequently the old dentist *thinks he* must come down in order to retain his patients. Now here he makes his first mistake. In the first place when a new man starts in a town, the old dentists, instead of calling upon him, and treating him as a brother dentist, consider him an interloper, they shun him, and show no fraternal feeling towards him. He feels hurt and determines he will "cut" prices in order to ruin their business and build up his own; the cutting process goes on all around until none of them are even making a fair living. You will say, "how is this to be overcome?" I will answer you that. When a person comes to your town to open an office, call on him, tell him your charges for operative and mechanical dentistry, and ask him to agree with you to a code of prices. If he has any professional honor he will comply. And when your code is made, *live up to it.* Make *no secret* contracts with your *patients*, for as soon as you have done that you have broken your word of honor. Your competitor will surely hear of it, and then lose confidence in you, and you certainly can not complain if he cuts you in prices. You had better fill one cavity a day for three dollars, than two cavities at one dollar and a half each, as you have less work for the same pay, besides honoring your professional skill. And the public think higher of a man charging good prices than of one who will do the same work cheaply. Should your competitor decline to make any code of prices with you, do not lower your prices, for then you confess to be no better operator than he. Keep your prices up, and if your patients object to them, you have only to tell them you do not do *cheap* work; they will soon see that you are the better of the two, and your business will increase rather than diminish.

If you charge three dollars for a filling your competitor charges only one dollar for, he has to do three times as much work as you for the same amount of money. It is a mistaken idea to think people will *not* pay good prices, They will do it, and they *do* do it when you tell a person the price will be so much. Keep to it. Do not reduce it. And you will soon find you are doing, if not a large, a remunerative practice. Look around you, and you will see it is not those who are doing the largest business who are making the most money, but those who are getting the best prices for their services. Keep your prices up. Gentlemen, do your work well, and you will soon find you are becoming richer ; you will be honoring your profession, and gaining a reputation. C.

